# The mediating role of organizational trust in the impact of digital leadership on job performance and job satisfaction

**DOI:** 10.3389/fpsyg.2026.1879796

**Published:** 2026-08-13

**Authors:** Kazım Kaya, Ziya Bahadır, Keziban Yoka, Ahmet Yalçınkaya

**Affiliations:** 1 Faculty of Sports Sciences, Kırşehir Ahi Evran University, Kırşehir, Türkiye; 2 Faculty of Sports Sciences, Recreation, Erciyes University, Kayseri, Türkiye; 3 Institute of Social Sciences, Niğde Ömer Halis Demir University, Niğde, Türkiye

**Keywords:** digital leadership, job performance, job satisfaction, organizational trust, public sector

## Abstract

This study examines the effect of digital leadership on job performance and job satisfaction, focusing on the mediating role of organizational trust. Utilizing a quantitative research design, data were collected via an online survey from 443 public employees working within the Ministry of Youth and Sports across Kırşehir, Nevşehir, Yozgat, and Aksaray. The data were analyzed using partial least squares structural equation modeling. The measurement model demonstrated satisfactory reliability and validity. Structural model testing and bootstrap analysis revealed that digital leadership has a significant and positive effect on job performance, whereas its direct effect on job satisfaction is not significant. Furthermore, digital leadership significantly influences organizational trust, which in turn exerts strong, positive effects on both job performance and job satisfaction. Consequently, organizational trust partially mediates the relationship between digital leadership and job performance, and fully mediates the relationship between digital leadership and job satisfaction. These findings underscore that the impact of digital leadership on employee outcomes is heavily contingent upon trust-building mechanisms. This research provides actionable insights for public sector policymakers and human resource managers aiming to leverage digital leadership strategies to cultivate organizational trust, thereby optimizing employee outcomes in digitally transforming environments.

## 1 Introduction

In contemporary organizations, leadership is defined as a multidimensional phenomenon involving directing, influencing, and supporting individuals or groups toward common goals. This concept, which plays a critical role in achieving organizational success, is not limited to individual characteristics and skills but also encompasses the ability to motivate human resources and strategically direct organizational activities in order to achieve organizational goals (Birami et al., 2025). Indeed, leadership, particularly in educational and organizational contexts, encompasses multifaceted responsibilities including effective process management, employee guidance, and the integration of technological advancements into organizational structures (Blömeke and Klein, 2013). Accordingly, effective leaders are expected to unite their teams around common goals, motivate employees, and provide inspiring guidance (Altıntaş and Merdan, 2025). Current studies reveal that leadership is not solely composed of individual competencies; it is also a dynamic and multidimensional process shaped by organizational structure, cultural values, and environmental factors (Durmaz and Yılmaz, 2024). This transformation has become even more pronounced, particularly with digitalization. In recent years, the rapid development of digital technologies has fundamentally changed the way businesses operate, reshaping not only production processes but also customer relationships, marketing strategies, and decision-making mechanisms (Brynjolfsson and McAfee, 2014). In this context, digital transformation is considered not only a technological necessity but also a strategic element that provides a sustainable competitive advantage for today’s organizations (Westerman et al., 2011). Emerging alongside these developments, digital leadership is a leadership approach that strategically manages the digitalization process and enables organizations to effectively operate in the digital age. Digital leaders assume critical responsibilities, including developing innovative solutions, increasing organizational agility, and enhancing employees’ digital competencies (Güler, 2023). However, this leadership approach plays a key role in implementing digital strategies by making business processes more flexible and agile (Önbıçak and Akkoyun, 2022). Furthermore, digital leadership encompasses not only the effective use of technological tools but also the adaptation of organizational culture to digital transformation and the active participation of employees in this process (Avcı and Turunç, 2012; Brunner et al., 2023; Camp et al., 2022). Digital leadership, unlike traditional leadership approaches, prioritizes data-driven decision-making processes and encourages the effective use of tools such as artificial intelligence, data analytics, and cloud technologies (Saarikko et al., 2020). Despite the growing body of research on digital leadership, its direct relationship with job satisfaction remains inconsistent in empirical findings, particularly in public sector contexts. Moreover, limited studies have sufficiently explained the psychological mechanism through which digital leadership translates into employee attitudes in bureaucratic organizations. In addition, the mediating role of organizational trust in this relationship has not been adequately examined within the framework of digital transformation in government institutions. Accordingly, managing digital transformation in the public sector is of strategic importance due to its unique bureaucratic and cultural dynamics. The Ministry of Youth and Sports, which constitutes the population of this research, offers a highly unique and well-reasoned ground for examining digital leadership in the public sector. Unlike other state institutions that traditionally have hierarchical and rigid bureaucratic structures, the Ministry of Youth and Sports is responsible for managing a comprehensive digital service network that directly addresses a dynamic, flexible, and young population, including nationwide networks of youth centers, digital event platforms, and centralized sports information systems. However, despite this dynamic job description, the fact that internal operations are based on established and traditional public legislation can create a certain resistance and adaptation friction among personnel during the transition from face-to-face supervision to digital and remote work environments. Therefore, the success of technological transformation within the ministry depends not only on infrastructure investments but also on the presence of digital leaders who can manage this process in a flexible and visionary way. This contributes to faster decision-making processes and increased organizational efficiency, while also creating environments for continuous learning and development to enhance employees’ digital skills (Bharadwaj et al., 2013). Especially during transitional periods when rigid bureaucratic institutions migrate to digital work environments, organizational trust becomes a critical structural asset rather than a luxury. Another important concept that stands out in digital transformation processes is organizational trust. Organizational trust refers to the trust employees have in their managers and the organization, and their perception that the organization is transparent, honest, and supportive (Yılmaz and Altınkurt, 2012). When employees both trust their managers and feel trusted, it contributes to the development of positive attitudes towards their work, which in turn, has positive effects on outcomes such as job performance, job satisfaction, and organizational commitment (Dirks and Ferrin, 2001).

In addition, organizational trust contributes to reducing negative situations such as work stress and burnout. More broadly, organizational trust encompasses a multidimensional structure ranging from interpersonal relationships to inter-organizational interactions (Li, 2008). In the age of digitalization, the importance of organizational trust is increasing, and with the widespread adoption of remote work and virtual teams, leaders’ trust-building skills are becoming critical. The effective use of digital tools, data security practices, and transparent communication processes plays a decisive role in building employee trust in the organization (Coronado-Maldonado and Benítez-Márquez, 2023). Furthermore, organizational trust contributes to a faster and more effective digital transformation by reducing employee resistance during change processes (Dogra et al., 2024). When evaluated in terms of organizational outcomes, job performance is an important indicator expressing the extent to which employees perform their tasks effectively and efficiently. Performance is shaped by the interaction of an individual’s competencies, motivation level, and environmental conditions (Rivai, 2006), and reflects the degree to which goals are achieved at both individual and organizational levels in both quantitative and qualitative dimensions (Güner et al., 2008; Shahzadi et al., 2014). However, job satisfaction is defined as the positive emotional attitude that employees develop towards their work and directly affects employee motivation, commitment, and productivity (Baş and Ardıç, 2002). Employees with high levels of job satisfaction are seen to approach their tasks with greater responsibility and contribute more strongly to organizational goals (Candan, 2013). Recent studies increasingly highlight the impact of digital leadership on employee behavior and organizational outcomes. These studies demonstrate that digital leadership is a holistic approach encompassing not only the effective use of technology but also fostering innovation, increasing organizational flexibility, and enabling employee adaptation to digital transformation processes (Zhao, 2024; Farag, 2025; Fiteriani and Rahmadani, 2025). Although the concepts of digital leadership, organizational trust, and employee outcomes have been individually discussed in the literature, a significant research gap remains regarding how these variables interact holistically within a single structural model. Specifically, despite growing recognition of digital leadership, the precise underlying socio-psychological mechanisms through which it translates into heightened job satisfaction and job performance are not yet fully understood. Recent studies have highlighted the growing importance of digital leadership and organizational trust in facilitating employee adaptation and organizational effectiveness during digital transformation; however, evidence regarding the underlying mechanisms remains limited (Altıntaş et al., 2024; Kicheva-Kirova, 2025; Ying et al., 2025). Previous studies often overlook the strategic mediating role of organizational trust, creating a fragmented understanding of leadership-employee dynamics in digitally transforming environments. Furthermore, organizational trust has long been recognized as a key determinant of positive employee attitudes and workplace outcomes (Iverson and Deery, 1997; Sabuncuoğlu and Tak, 2001). To address this gap, this study provides a dual theoretical contribution by integrating Transformational Leadership Theory and Social Exchange Theory (SET) as complementary lenses. Transformational Leadership Theory explains how digital leaders exhibit a visionary role model in a virtual environment, intellectually stimulate employees, and lead digital change (input). On the other hand, understanding why and through what mechanism employees respond to this style with positive outcomes is driven by Social Exchange Theory (SET). From the SET perspective, when transformational digital leaders offer employees autonomous workspaces and support them in the digital transition, a positive social exchange process is triggered (). Employees perceive this leadership support as an organizational investment, which fosters organizational trust. According to the norm of reciprocity, this trust is repaid to the organization through positive psychological and behavioral feedback, namely higher job satisfaction and increased job performance. Consequently, this study fills a crucial literature gap by offering a unified model that explains both leadership inputs and the cognitive-behavioral outputs of employees. In terms of generalizability, the findings of this research extend beyond the specific sample context, offering scalable insights for various modern industries undergoing digital transformation. By demonstrating that organizational trust serves as a universal relational bridge, this model provides actionable evidence for diverse organizational structures aiming to optimize employee performance and well-being in the digital era. Ultimately, this study aims to comprehensively explain the structural effects of digital leadership behaviors on employee trust, job performance, and job satisfaction.

## 2 Literature review and theoretical development

### 2.1 Digital leadership

While leadership was initially defined by Fiedler (1967) and Stogdill (1948) in terms of individuals’ personal characteristics and their capacity for group guidance, later studies () explained leadership through behavioral patterns dependent on situational conditions rather than fixed traits; in this context, the understanding of leadership has undergone significant evolution throughout history. Thus, while charismatic leadership based on innate qualities was prominent before 1940 (Stogdill, 1948; Fiedler, 1967), approaches emphasizing leadership behavior patterns came to the forefront during the 1940–1960 period (Blake and Mouton, 1964; House and Mitchell, 1975). During the 1960–1980 period, relational and situational leadership emerged, and the voluntary relationships that leaders establish with their followers became more important (House and Mitchell, 1975; Hersey and Blanchard, 1969; Stogdill, 1948; Fiedler, 1967; Westerman et al., 2014; Tukiyo et al., 2017). Today, with Industry 4.0, digitalization and visionary leadership have come to the forefront, adopting a responsive and multi-dimensional leadership approach (Raza, 2016; Öz, 2020). When technological developments are integrated, it is seen that the virtual world and the real world can merge and form a different kind of whole; the ability to guide and effectively adapt to these processes of change and transformation is defined in the literature with the concepts of Leadership 4.0 or digital leadership (Prince, 2017; Yusof et al., 2019; Sheninger, 2014). Digital leadership is conceptualized as extending beyond organizational management and technology use, being directly related to employees’ psychological processes. From the perspectives of organizational and occupational psychology, studies examine the impact of leaders on psychological outcomes, including employees’ perceptions of digital self-efficacy, work motivation, job satisfaction, psychological trust, and job commitment, in a digitized work environment. For example, it has been shown that digital leadership positively affects employees’ job commitment and perceptions of digital self-efficacy, which in turn is reflected in employee engagement and job performance (Ragu-Nathan et al., 2008). Furthermore, it has been shown that individual perceptions, such as psychological job design and organizational fit, play important mediating roles in the relationship between digital leadership and employee creativity (Zhu et al., 2022). In this context, digital leadership is considered not only as a process of regulating interaction processes through information and communication technologies, but also as a process that improves employees’ psychological well-being and organizational attitudes (Şahin et al., 2020). Although there are different definitions of digital leadership, across all the disciplines studied, digital leaders are commonly described as technologically adept and as driving the digital transformation of the organization and changes in organizational culture (López-Figueroa et al., 2025). Digital leadership is described as having two dimensions: digital literacy, digital competencies, and higher leadership competencies encompassing behaviors and the management of digital transformation (Zeike et al., 2019; Bingöl, 1993).

### 2.2 Job performance

Performance is defined as the extent to which a job is accomplished under specific conditions or the level of behavior exhibited by an employee (Bingöl, 1993). Performance appraisal is a process that systematically evaluates employees’ current performance and future potential against predetermined criteria. The widespread use of performance appraisal results in human resources management practices such as promotion, training, and development, as well as compensation, has led to this topic being extensively studied in academic and applied research in recent years. Job performance serves as a means for an individual to achieve defined goals related to a job, role, or organization; however, the tangible results of these goal-oriented behaviors become apparent during the execution of the job. Job performance is considered a multi-dimensional and complex activity rather than a single action. According to Jacobs et al. (2013), job performance is a distinct behavioral phenomenon that should be considered independently of job outcomes, such as success or productivity (Jacobs et al., 2013). The literature states that job performance consists of different dimensions. These dimensions include core task performance, creativity, participation in and performance level in training programs, organizational citizenship behaviors, and safety performance. Work behaviors that harm organizational goals, such as workplace aggression, substance abuse at work, tardiness, and absenteeism, are also considered among the dimensions taken into account in evaluating job performance (Ng and Feldman, 2008).

### 2.3 Job satisfaction

Job satisfaction is an important concept that reflects an individual’s level of contentment with their work and directly affects employees’ psychological, social, and professional lives. High job satisfaction increases employee morale and motivation, helping them establish healthier, more constructive relationships with their colleagues. This situation positively affects not only individual performance but also team cohesion and organizational efficiency (Eren, 2004; Tütüncü, 2000). Job satisfaction is an evaluation process reflecting an employee’s level of contentment with their work (Froese and Xiao, 2012), and is based on individual perceptions of both job characteristics (nature of the job, management style) and job outcomes (salary, job security). In this context, job satisfaction can be defined as the totality of emotional responses that an employee develops in line with their perceptions of their job and working conditions (Çekmecelioğlu, 2005). Therefore, positive emotions arising from meeting an individual’s expectations and needs form the basis of job satisfaction (Yılmaz, 2020; Yalçınkaya et al., 2025). In the literature, job satisfaction is also considered as a whole of an individual’s general attitudes and feelings towards work, and is evaluated as a reflection of personal value judgments regarding the gains and rewards obtained from work (). Accordingly, how employees perceive and evaluate their jobs plays a fundamental role in determining their job satisfaction levels. It has been observed that employees experience higher levels of satisfaction in work environments that allow them to utilize their individual competencies, are diverse, present a moderate level of challenge, and provide a certain degree of autonomy (Olusegun, 2013). This situation demonstrates that job satisfaction is related not only to the structural characteristics of the job but also to the meaning the employee assigns to their work experience. Indeed, job satisfaction is considered a multidimensional construct encompassing emotional evaluations of different aspects of the job and directly linked to the extent to which organizations meet the needs of their employees (Efraty and Sirgy, 1990). Conversely, employees are likely to experience disappointment and develop job dissatisfaction if their expectations are not met in the work environment. This situation is closely related to the degree of alignment between the employee’s expectations and the conditions offered in the work environment. An increasing gap between expectations and actual gains leads to a decrease in job satisfaction levels (Lam et al., 2001). In conclusion, job satisfaction is an important indicator of employees’ level of adaptation to their jobs and organizations; it is shaped by the attitudes and behaviors individuals develop in response to their needs and expectations being met. Employees who feel that their efforts are being rewarded and that they are supported experience higher levels of job satisfaction; this contributes positively to the development of both individual performance and organizational relations and work outcomes (Babalola, 2016).

### 2.4 Organizational trust

Trust is a fundamental element in establishing and maintaining interpersonal relationships; it is a multifaceted concept of common interest to various disciplines such as sociology, economics, psychology, and management sciences. However, researchers from different disciplines have defined trust in various ways, according to their own theoretical frameworks. While studies in the field of psychology address trust in the context of the attitudes and personal characteristics of individuals who trust and are trusted, sociological approaches evaluate trust at the level of social integration and systems. In economic literature, trust is examined primarily in the context of calculable risks and institutional structures (Lewicki and Bunker, 1996). Furthermore, current organizational psychology research reveals that trust has behavioral and relational dimensions such as communication, transparency, and authenticity, and exhibits a multidimensional structure in an organizational context (Fischer et al., 2023). This multidimensional structure demonstrates that trust plays a critical role in an organizational context. Organizational trust is a multidimensional phenomenon of organizational psychology that plays a crucial role in enabling employees to develop a sense of psychological security and exhibit positive attitudes in their interactions within the organization. Indeed, research shows that high levels of organizational trust and a sense of unity of purpose positively influence both psychological and behavioral outcomes such as job satisfaction, productivity, intention to stay in the job, and overall life satisfaction (Johannsen and Zak, 2021). In addition, it has been reported that the level of trust employees perceive shapes their attitudes and behaviors; individuals exhibit more pro-social behavior and develop more constructive attitudes in organizational relationships in high-trust environments (Zhang and Ma, 2025). These findings reveal that organizational trust is a significant determinant not only of interpersonal relationships but also of key organizational outcomes such as job satisfaction, commitment, and performance. When examining the development of the concept of organizational trust, it is seen that its origins lie in organizational psychology and organizational behavior studies of the 1930s. In particular, McGregor’s (1960) Theory X and Theory Y represent one of the earliest frameworks emphasizing the role of trust in organizational settings. Subsequently, Berger and Luckmann’s (1966) social construction of reality highlighted how trust is socially produced and sustained through shared meanings and interactions within institutional contexts. Blau’s (1964) social exchange theory further advanced this understanding by arguing that fair, supportive, and consistent organizational behaviors activate the norm of reciprocity, thereby facilitating the emergence of trust-based relationships. In addition, conceptualized interpersonal trust as an expectancy that the word, promise, or statement of another individual can be relied upon, underscoring trust as a foundational element of interpersonal relations (Saracel et al., 2023). In this context, organizational trust is considered a construct that develops over time as an individual’s expectation that the organization will not harm them and will act in a supportive manner strengthens. Since the 1980s, organizational trust has received increasing attention in the field of organizational behavior and has become one of the main topics of research (Yalçınkaya et al., 2025).

### 2.5 Relationship between concepts

Digital leadership is a multidimensional concept that has been defined in various ways in the literature. El Sawy et al. (2016, p. 142) conceptualize digital leadership as the capability of “doing the right things” to ensure the strategic success of digital transformation within organizations and broader business ecosystems. Similarly, Sheninger (2014, p. 2) describes digital leaders as individuals who set direction, influence others, initiate sustainable change through effective use of information, and anticipate future transformations. In addition, Westerman et al. (2014) define digital leadership as a leadership approach that enables organizational mobilization through digital awareness, competence, and influence. Collectively, these definitions emphasize that digital leadership extends beyond technological competence, encompassing strategic vision, influence, and the capacity to guide organizational transformation in digitally driven environments.

**Figure 1:**
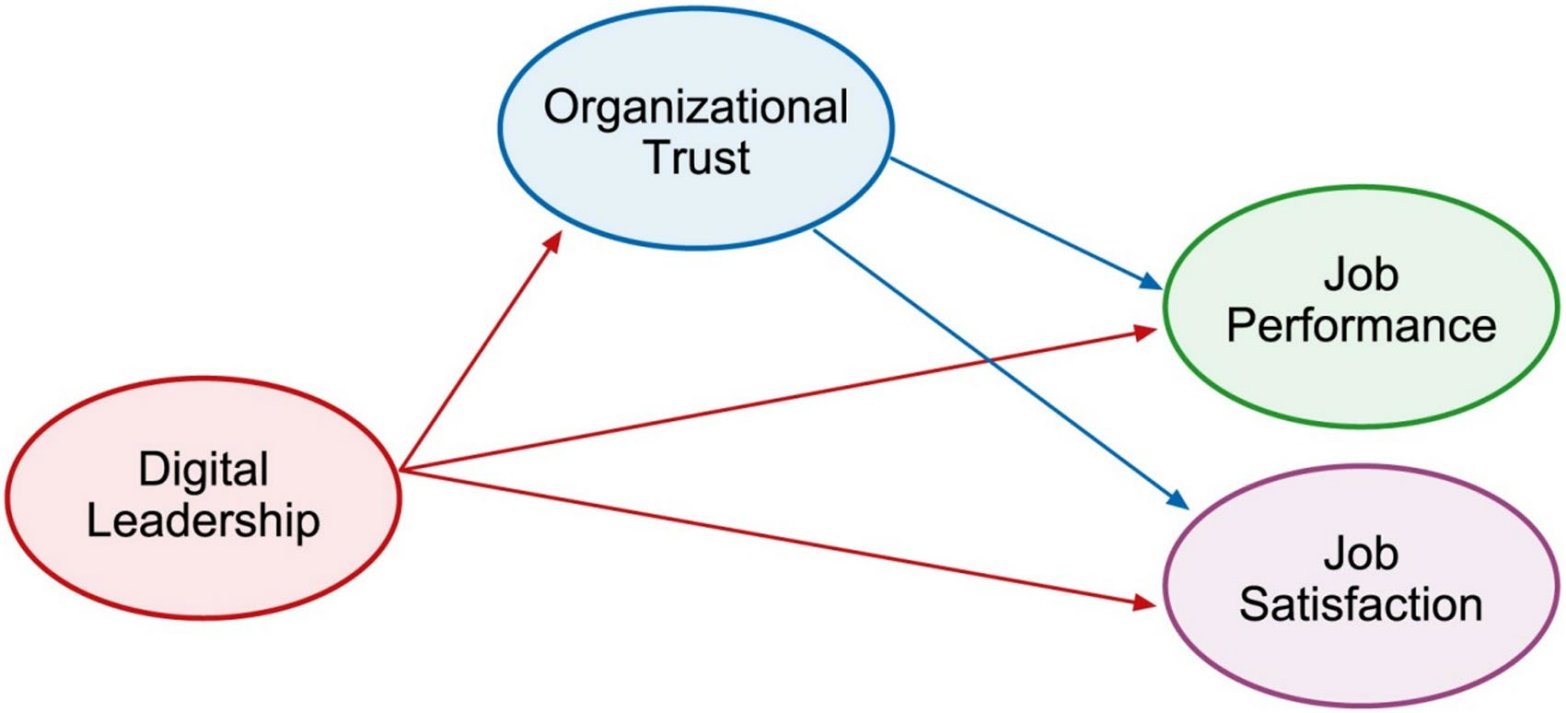
Research model.

These definitions show that digital leadership is not limited to technology; it also encompasses the ability to set strategic direction, manage transformation, and drive organizational impact. In this context, the relationship between digital leadership and job performance is explained by the capacity of organizations to enhance employees’ effectiveness in digital transformation processes (Niemi, 2025). Digital leaders are transforming the way we do business by encouraging employees to use technological tools effectively to streamline work processes. This transformation enables employees to work faster, more efficiently, and without errors, positively impacting their performance levels (Zeike et al., 2019). Therefore, digital leadership is considered a strategic leadership approach that plays a critical role in improving both individual and organizational performance. The impact of digital leadership on job satisfaction is largely shaped by employees’ perceptions and emotional evaluations. It is known that employees experience increased job satisfaction in work environments where they can develop themselves, participate in innovative practices, and feel supported (Froese and Xiao, 2012). At this point, digital leaders can increase job satisfaction by offering employees learning opportunities and fostering a participatory, supportive climate. However, the uncertainty and pressure for change brought about by digital transformation processes can negatively affect employees’ job satisfaction in some cases (Yalçınkaya et al., 2025). Therefore, the relationship between digital leadership and job satisfaction needs to be examined in both its direct and indirect dimensions, encompassing both its positive and potential negative impacts. Another critical element in achieving success in digital transformation at the organizational level is organizational trust. Organizational trust refers to the confidence employees have in their organization, managers, and colleagues; this trust directly affects employees’ motivation, commitment to work, and performance (Yılmaz and Altınkurt, 2012). In organizations where trust prevails, employees take on more responsibility, focus more on their work, and improve their performance (Huff and Kelley, 2003). Similarly, organizational trust contributes to employees developing positive feelings towards their work and increases job satisfaction (Yalçınkaya et al., 2025). Literature shows a strong relationship between digital leadership and organizational trust. Digital leaders, with their technological competencies and qualities such as transparency, ethical responsibility, and visionary thinking, increase employee trust in the organization and facilitate participation in change (Niemi, 2025; Huff and Kelley, 2003; Plokha and Murzha, 2025). The quality of leader-member interactions plays a critical role in reinforcing employees’ trust in their leader and the organization’s digital capabilities, especially in remote and virtual environments (). The theoretical basis for these relationships lies in social exchange theory and transformational leadership theory. According to social exchange theory, employees reciprocate the support and trust they receive from their leaders; this is reflected in higher job performance (Blau, 1964; Cropanzano and Mitchell, 2005). Transformational leadership describes digital leadership characteristics such as vision, inspiration, and support for individual development, and contributes to understanding how trust is reflected in behavioral outcomes (Bass and Avolio, 1994). In this context, organizational trust is expected to mediate the relationships between digital leadership and job performance and job satisfaction. The trusting environment created by digital leaders indirectly affects both employee performance and job satisfaction (Alsalman and Chyad, 2025; Wider et al., 2025). This situation contributes to the healthier functioning of organizational processes and increases employees’ commitment to the organization. Based on the literature, the following research model and hypotheses have been formulated (see Figure 1).

Drawing upon Transformational Leadership Theory and Social Exchange Theory, this study proposes that digital leadership functions as a strategic leadership approach that enhances organizational trust and subsequently improves employee outcomes. While Transformational Leadership Theory explains how leaders influence employees through vision, inspiration, and support, Social Exchange Theory explains why employees reciprocate these leadership behaviors with greater trust, satisfaction, and performance. Accordingly, the following hypotheses are proposed.

*H1*: Digital leadership has a significant impact on job performance.

*H2*: Digital leadership has a significant impact on job satisfaction.

*H3*: Digital leadership has a significant impact on organizational trust.

*H4*: Organizational trust has a significant impact on job performance.

*H5*: Organizational trust has a significant impact on job satisfaction.

*H6*: Organizational trust mediates the effect of digital leadership on job performance.

*H7*: Organizational trust mediates the effect of digital leadership on job satisfaction.

## 3 Materials and methods

### 3.1 Model of the research

This research follows a correlational and cross-sectional design within the quantitative research paradigm. Correlational research design is a descriptive method that does not involve experimental intervention and allows the examination of the direction, strength, and significance of relationships between two or more variables (Creswell and Creswell, 2018; Karasar, 2022). The study utilized Structural Equation Modeling (SEM), which enables the simultaneous testing of direct and indirect relationships among variables. SEM is a, robust statistical technique used to examine and test relationships between observed and latent variables by considering both the measurement and structural models holistically (Kline, 2015).

### 3.2 The population and sample of the study

The population of this research consists of employees working in units affiliated with the Ministry of Youth and Sports in Türkiye. The study sample consists of employees working in the provinces of Kırşehir, Nevşehir, Yozgat, and Aksaray. The inclusion of these provinces in the research was chosen both to increase the feasibility of the data collection process and to obtain a more representative sample by encompassing the views of public employees working in different provinces. To determine the appropriate sample size for the study, an *a priori* power analysis was conducted using G*Power 3.1 software. In PLS-SEM studies, the minimum sample size is determined by the maximum number of paths leading to any endogenous variable in the model (Hair et al., 2014). In this study, the maximum number of paths to any dependent variable in the structural model was 2. Accordingly, in the G*Power analysis, the “Linear Multiple Regression: Fixed model, *R*^2^ increase” test was selected under the F-tests; the moderate effect size (*f*^2^ = 0.15), Type I error rate (*α* = 0.05), statistical power level (1 − *β* = 0.99), and number of predictor variables (k = 2) were taken into consideration (Chathoth et al., 2007; Pillai et al., 1999). The analysis determined that the minimum sample size required is 146. The sample size used in the study met this requirement, indicating a high statistical power (99%) and sufficient sensitivity in the analyses conducted.

“In this study, convenience sampling, a non-probability sampling method, was utilized to reach public sector employees working under the Ministry of Youth and Sports across the designated provinces. This approach was selected due to its practical efficiency, geographical accessibility, and the large organizational layout of the ministry. Unlike the initial categorization, criteria such as voluntary participation and the complete submission of survey forms were treated strictly as ethical prerequisites and standard data-cleaning protocols, rather than theoretical sampling criteria (). A total of (443) valid responses were retained for the final analysis after removing incomplete or patterned responses.”

Table 1 presents the demographic information of the participants in the study.

**Table 1:** Socio-demographic information of participants.

Variable	Category	*N*	%
Gender	Male	247	55.8
	Woman	196	44.2
Age	20–25 ages	31	7.0
	26–31 ages	78	17.6
	32–37 ages	104	23.5
	38–43 ages	106	23.9
	44 age and over	124	28.0
Marital status	Single	120	27.1
	Married	323	72.9
Educational level	High School	129	29.1
	College	67	15.1
	University	199	44.9
	Under graduate	48	10.8
Working seniority	1–5 year	158	35.7
	6–10 year	98	22.1
	11–15 year	91	20.5
	16–20 year	47	10.6
	21 year and over	49	11.1
Monthly income	20.001–40.000 TL	28	6.3
	40.001–60.000 TL	230	51.9
	60.001–80.000 TL	112	25.3
	80.001–100.000 TL	60	13.5
	100,001 TL and over	13	2.9

### 3.3 Data collection tools

The survey form used as a data collection tool in the research consists of five sections. The first section includes questions aimed at determining the demographic characteristics of the participants (e.g., age and gender). The second section consists of statements designed to measure digital leadership, the third section job performance, the fourth section job satisfaction, and the fifth section organizational trust. Data were collected online using Google Forms. The survey link was distributed to employees working under the Ministry of Youth and Sports in the selected provinces through institutional communication networks and professional contacts. Participation was entirely voluntary, and no administrative mandate or compulsory instruction was used during the data collection process. Prior to completing the survey, participants were informed about the purpose of the study, the confidentiality of their responses, and their right to withdraw at any stage. Informed consent was obtained from all participants to ensure compliance with ethical principles. To reduce potential Common Method Bias (CMB), participants were assured that their responses would remain anonymous and confidential and that the collected data would be used solely for scientific purposes. Furthermore, the questionnaire was organized into separate sections corresponding to each construct (digital leadership, job performance, job satisfaction, and organizational trust), thereby minimizing the likelihood of respondents developing automatic response patterns or cognitive consistency effects during the completion process. After excluding incomplete and invalid responses, a total of 443 valid questionnaires were retained for analysis.

#### 3.3.1 Digital leadership scale

To measure digital leadership, the Digital Leadership Scale developed by Zeike et al. (2019) and adapted into Turkish by Sürücü et al. (2022) was used (Zeike et al., 2019). The scale is one-dimensional and comprises 6 items. Participants rate the statements using a 5-point Likert scale.

#### 3.3.2 Job performance scale

To measure employees’ job performance, the 4-item Employee Performance Scale developed by Sigler and Pearson (2000) and adapted into Turkish by Çöl (2008) was used (Çöl, 2008). The Cronbach’s alpha coefficient of the scale was reported as 0.828.

#### 3.3.3 Job satisfaction scale

To measure job satisfaction levels, the scale developed by Judge et al. (1998) and adapted into Turkish by Başol and Çömlekçi (2020) was used (Başol and Çömlekçi, 2020). The scale is one-dimensional and consists of 5 items in total. An example statement is “I am quite satisfied with my current job.” The Cronbach’s Alpha coefficient of the scale was stated as 0.88.

#### 3.3.4 Organizational trust scale

Organizational trust was measured using the 17-item scale developed by Çalışkan (2021). The original scale comprises three dimensions: trust in coworkers (7 items), trust in management (5 items), and trust in the organization (5 items). During the measurement model assessment, all 17 items were initially included. Following the recommendations of Hair et al. (2022), items with inadequate psychometric properties were removed based on their outer loadings and their effects on composite reliability and convergent validity. Consequently, the final measurement model included nine empirically validated items.

### 3.4 Analysis of data

In this study, the Partial Least Squares Structural Equation Modeling approach was used to examine the relationships between variables. PLS-SEM is a powerful method, especially for analyzing complex models, when sample size is limited, and when the data distribution does not meet the normality assumption (Hair et al., 2014; Chathoth et al., 2007). The research primarily focused on evaluating the measurement model. In this phase, reliability and validity analyses of the scales used were performed. Internal consistency reliability was assessed using Cronbach’s alpha and composite reliability (CR) coefficients, while mean variance explained (AVE) values were examined for convergent validity. The fact that the relevant values are within acceptable limits indicates that the measurement model has sufficient reliability and validity (Hair et al., 2017).

In addition, discriminant validity was evaluated by considering the Fornell-Larcker criterion and the HTMT ratio. In this context, it is expected that the AVE root mean of each construct will be higher than its correlations with other constructs, and that the HTMT values will remain below the recommended thresholds. The validity and reliability analysis results for the measurement model are presented in Table 2. Following the validation of the measurement model, structural model analysis was performed. In the structural model, path coefficients between variables, explained variance (*R*^2^) values, and effect sizes (*f*^2^) were examined. The predictive power of the model was evaluated using the *Q*^2^ value. The Bootstrap method was used to test the mediating effect in the study. Within the scope of Bootstrap analysis, the significance of indirect effects was tested using a resampling method (typically 5000 subsamples). If the confidence intervals do not include a zero value, the mediating effect is considered statistically significant (Hair et al., 2014; Preacher and Hayes, 2004).

**Table 2:** Validity indices and descriptive statistics of the scales.

Variable	Factor loading values	Ave.	St. deviation	VIF	CA	rho_A	CR	AVE
DL2	0.754	2.871	0.969	1.683	0.853	0.870	0.894	0.628
DL3	0.754	3.343	0.931	1.735				
DL4	0.816	2.774	1.018	1.898				
DL5	0.855	3.115	1.059	2.318				
DL6	0.777	3.045	0.918	1.948				
JP1	0.848	4.366	0.761	2.198	0.867	0.867	0.909	0.715
JP2	0.863	4.174	0.792	2.452				
JP3	0.861	4.099	0.803	2.282				
JP4	0.809	4.144	0.779	1.723				
JS1	0.768	4.305	0.891	1.721	0.871	0.880	0.912	0.722
JS2	0.854	3.682	1.094	2.172				
JS3	0.889	4.111	0.964	2.699				
JS5	0.882	3.953	0.955	2.668				
T9	0.816	3.973	0.969	3.002	0.937	0.938	0.947	0.667
T10	0.851	3.740	1.070	4.551				
T11	0.820	3.603	1.090	3.675				
T12	0.863	3.767	1.059	3.894				
T13	0.840	3.826	1.030	2.929				
T14	0.792	3.772	1.062	2.499				
T15	0.799	3.801	1.052	2.897				
T16	0.841	3.729	1.060	3.291				
T17	0.720	3.907	0.928	1.742				

DL, Digital Leadership; JP, Job Performance; JS, Job Satisfaction; T, Organizational Trust.

## 4 Findings

As shown in Table 2, the validity and reliability results of the measurement instruments are presented. The values of composite reliability (CR), average variance extracted (AVE), Cronbach’s alpha, rhoA, and VIF are within acceptable thresholds suggested in the literature, providing evidence for convergent validity (Hair et al., 2017). In addition, discriminant validity was assessed using the Fornell-Larcker criterion and the heterotrait-monotrait (HTMT) ratio (Karasar, 2022; Yıldırım and Şimşek, 2018). The results are presented in Table 3.

**Table 3:** Fornell–Larcker criterion and heterotrate–monotrate ratio (HTMT) values.

Fornell–Larcker criterion					Heterotrait–monotrait ratio (HTMT)				
	1	2	3	4		1	2	3	4
DL	0.792				DL				
JP	0.209	0.846			JP	0.237			
JS	0.156	0.620	0.850		JS	0.178	0.718		
T	0.208	0.437	0.579	0.817	T	0.222	0.477	0.634	

DL, Digital Leadership; JP, Job Performance; JS, Job Satisfaction; T, Organizational Trust.

In this study, the original 17-item Organizational Trust Scale developed by Çalışkan (2021) was initially included in the analysis. The measurement model was evaluated using SmartPLS, and its psychometric properties were assessed based on outer loadings, CR, AVE, and discriminant validity criteria. The results indicated that eight items showed low outer loadings and negatively affected the reliability and validity of the construct. Following the recommendations of Hair et al. (2022), these items were removed, resulting in a final scale consisting of nine items. The analyses were therefore conducted using the retained items. The removal of items was based solely on statistical criteria and was not guided by theoretical considerations. All latent constructs (digital leadership, organizational trust, job performance, and job satisfaction) were specified as reflective measurement models. This specification is appropriate because the observed indicators are conceptualized as manifestations of the underlying latent constructs. Accordingly, changes in the latent construct are expected to be reflected in all indicators, which are assumed to be highly correlated and interchangeable. Therefore, all constructs in this study were modeled as reflective in accordance with established PLS-SEM guidelines (Hair et al., 2019, 2022; Jarvis et al., 2003; Coltman et al., 2008).

Table 3 presents the Fornell-Larcker and HTMT criterion values. While the Fornell-Larcker criterion values are in agreement with the literature, HTMT values below 0.90 indicate that discriminant validity has been achieved (Mayer et al., 1995). Table 4 shows the goodness-of-fit values obtained from the model.

**Table 4:** Goodness-of-fit values.

	Saturated model	Estimated model
SRMR	0.058	0.088
d_ULS	0.859	1.958
d_G	0.392	0.458
Chi-Square	1.008.303	1.127.344
NFI	0.847	0.829

**Figure 2:**
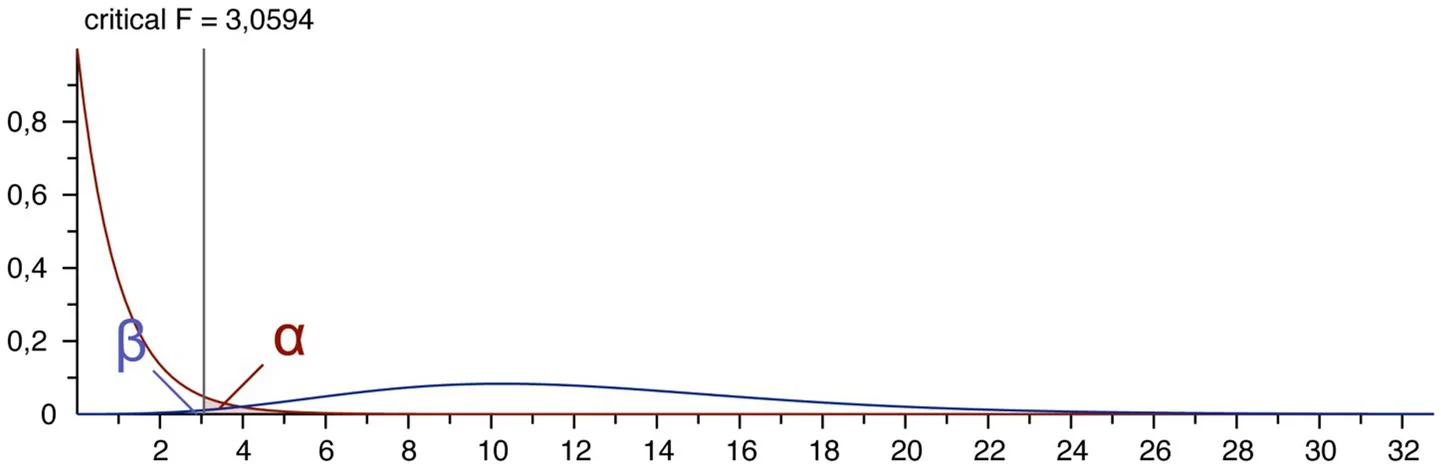
G-power analysis.

Table 4 shows that the goodness-of-fit values obtained from the established model are achieved (Brynjolfsson and McAfee, 2014). Although there are specific ranges in the literature for SRMR, chi-square, and NFI values, no distinction has yet been made for d_ULS and d_G values (Hair et al., 2017). Figure 2 shows the research model diagram.

**Figure 3:**
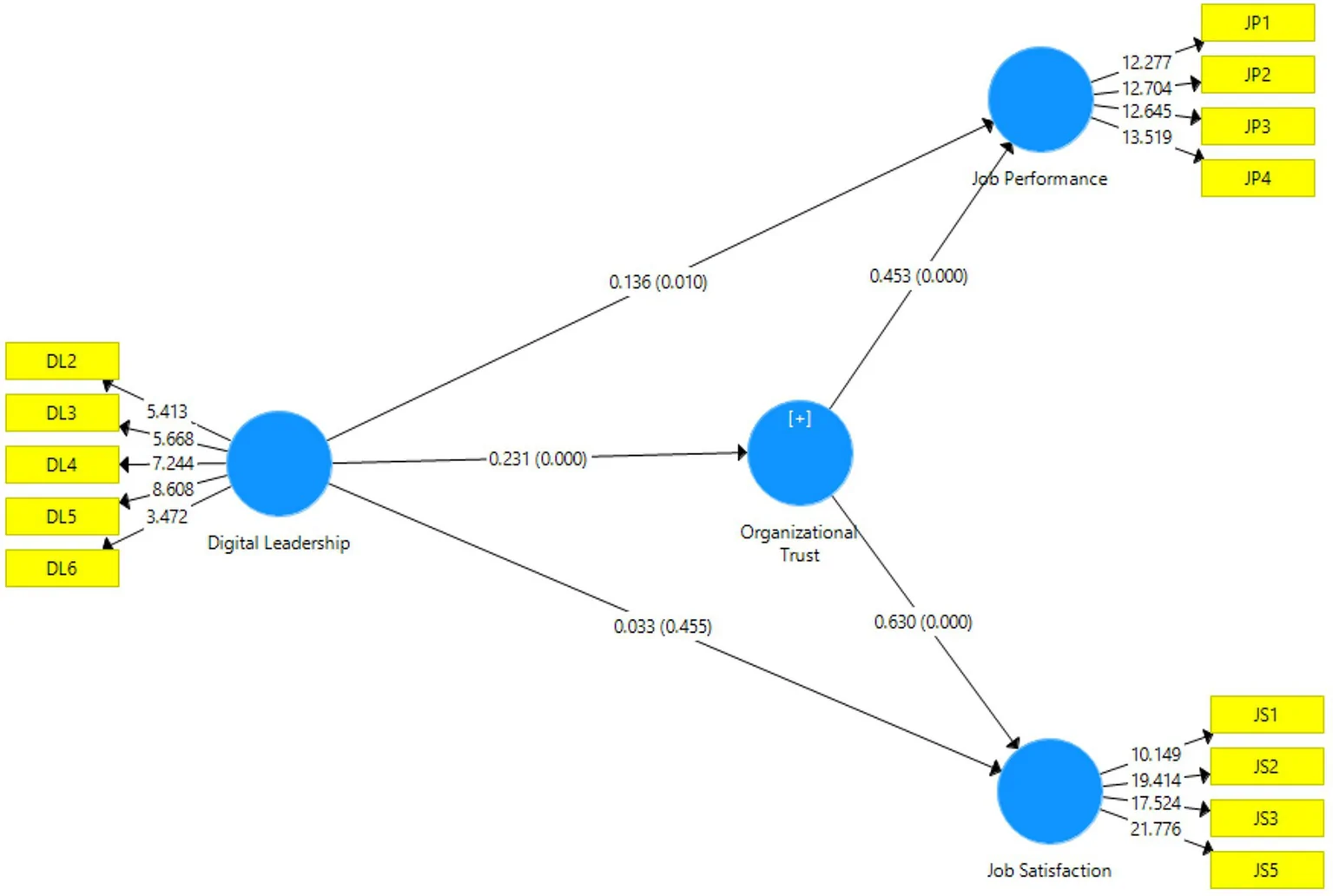
Total and indirect effects.

Although the NFI value obtained in this study was slightly below the conventional 0.90 threshold, recent PLS-SEM literature emphasizes that global model fit indices such as NFI should be interpreted cautiously in variance-based SEM. Rather than relying on NFI alone, the evaluation of PLS-SEM models should primarily focus on the measurement model quality, predictive relevance (*Q*^2^), explanatory power (*R*^2^), and the significance of structural path coefficients. Therefore, the NFI value should be considered together with these complementary model evaluation criteria rather than as a decisive indicator of model adequacy (Henseler et al., 2016) (see Figure 3).

Table 5 shows the structural equation model coefficients related to the mediating role.

**Table 5:** Hypothesis testing results.

	Std. beta	Beta	STDEV	*T* Statistics	*p*	2.5%	97.5%	*R*-square
DL → JP	0.136	0.140	0.053	2.566	0.010	0.038	0.236	0.206
DL → JS	0.033	0.037	0.044	0.747	0.455	−0.057	0.113	0.337
DL → T	0.231	0.236	0.058	3.994	0.000	0.108	0.337	0.043
T → JP	0.453	0.452	0.062	7.287	0.000	0.310	0.556	
T → JS	0.630	0.631	0.042	15.023	0.000	0.539	0.704	
DL → T → JP (Indirect)	0.105	0.106	0.028	3.737	0.000	0.053	0.162	
DL → T → JS (Indirect)	0.145	0.148	0.035	4.160	0.000	0.071	0.210	

DL, Digital Leadership; JP, Job Performance; JS, Job Satisfaction; T, Organizational Trust.

As shown in Table 5, the results of the mediation analysis examining the mediating role of organizational trust in the relationships between digital leadership and job performance, as well as job satisfaction, are presented. The significance of the direct and indirect effects was assessed using bootstrap analysis with 5,000 bootstrap resamples and percentile-based 95% confidence intervals. An indirect effect was considered statistically significant when the confidence interval did not include zero. The results indicate that digital leadership has a significant positive effect on job performance, whereas its direct effect on job satisfaction is not statistically significant. Organizational trust, however, has significant positive effects on both job performance and job satisfaction. Furthermore, the bootstrap analysis revealed that the indirect effects of digital leadership on both job performance and job satisfaction through organizational trust were statistically significant.

To determine the extent of the mediating effect, the Variance Accounted For (VAF) was calculated. According to Hair et al. (2022), VAF values below 20% indicate no mediation, values between 20 and 80% indicate partial mediation, and values above 80% indicate full mediation. The VAF value for job performance was 43%, indicating that organizational trust partially mediates the relationship between digital leadership and job performance. For job satisfaction, the VAF value was 81%. Together with the significant indirect effect and the non-significant direct effect, this finding indicates that organizational trust fully mediates the relationship between digital leadership and job satisfaction.

Although the coefficient of determination (*R*^2^ = 0.043) for organizational trust appears relatively low, this finding does not invalidate the mediation model. In PLS-SEM, *R*^2^ values are interpreted according to the complexity of the model, the nature of the endogenous construct, and the research context rather than by rigid cut-off values. Organizational trust is a multidimensional construct influenced by numerous organizational, interpersonal, and contextual factors beyond digital leadership alone. Therefore, it is theoretically reasonable that digital leadership explains only a limited proportion of the variance in organizational trust. More importantly, mediation analysis in PLS-SEM is based primarily on the statistical significance of the indirect effect rather than on the magnitude of the mediator’s *R*^2^. As emphasized by Hair et al. (2022), a significant indirect effect obtained through bootstrapping provides sufficient evidence for mediation, even when the explained variance of the mediator is relatively modest. Likewise, Ringle et al. (2024) argue that predictive relationships in PLS-SEM should be evaluated by considering path coefficients, indirect effects, predictive relevance (*Q*^2^), and the overall theoretical rationale together rather than relying solely on *R*^2^ values (Ringle et al., 2024). Therefore, the relatively low *R*^2^ for organizational trust reflects the multifactorial nature of the construct rather than a weakness of the proposed mediation model.

### 4.1 Discussion

The findings of this research reveal that digital leadership has a significant and positive impact on job performance, but its direct impact on job satisfaction is not significant (Table 5). This finding aligns with existing literature that emphasizes the impact of digital leadership on employees, particularly through outcomes such as performance (Turyadi et al., 2023; Wang et al., 2025), skills (Retnowati and Santosa, 2023), and creativity (Zhu et al., 2022). Indeed, digital leaders’ technology-focused management approach improves performance by accelerating business processes, enabling easier access to information, and enabling more systematic workflows. This finding aligns with studies indicating that digital leadership is particularly decisive for productivity and effectiveness (Zeike et al., 2019). However, it is stated that the effect of digital leadership on job satisfaction does not always occur directly but, in most cases, indirectly through intrinsic motivation or similar variables (Ayun and Erlin, 2025). Furthermore, it is emphasized that job satisfaction can mediate the relationship between digital leadership and job performance (Widyastuti et al., 2025). The perceived insignificant direct impact of digital leadership on job satisfaction opens up an important area of debate. Job satisfaction is considered a multidimensional construct, fueled not only by the structural characteristics of the job but also by the individual’s perceptions, expectations, and emotional evaluations (Froese and Xiao, 2012). Although digital transformation processes facilitate ways of doing business, they can also lead to negative perceptions for employees, such as uncertainty, increased workload, the need for continuous learning, and pressure to adapt to technology (Yalçınkaya et al., 2025). At this juncture, the continuous drive toward digital optimization led by digital leaders can inadvertently expose employees to structural stressors such as technostress and digital fatigue (Ragu-Nathan et al., 2008). While the implementation of new software and accelerated workflows directly boosts objective task performance, the underlying pressure to maintain constant digital connectivity and rapidly master shifting platforms can induce role anxiety. This cognitive strain effectively suppresses any direct, spontaneous increase in overall job satisfaction. From the perspective of Social Exchange Theory (SET) (Blau, 1964; Cropanzano and Mitchell, 2005), this modest yet non-negligible relationship between digital leadership and job satisfaction reflects an important socio-psychological dynamic within public sector organizations. In highly institutionalized and bureaucratic contexts, digital leadership initiatives are typically implemented through top-down directives accompanied by formal monitoring and control mechanisms. As a result, employees may initially interpret such digital transformation efforts as impersonal, rigid, and predominantly transactional rather than supportive or relational in nature. These initial perceptions may attenuate the immediate positive impact of digital leadership on job satisfaction, as employees often require time to reinterpret these initiatives as genuine forms of organizational support rather than mere administrative control. Consequently, digital leadership capabilities alone may not possess sufficient affective leverage to directly elicit intrinsic emotional outcomes such as job satisfaction. Within the framework of SET, effective social exchange relations depend on employees perceiving organizational actions as voluntary socio-emotional investments rather than obligatory structural requirements. When digital leadership is primarily experienced as a compliance-oriented mechanism, the reciprocal socio-emotional response that underpins job satisfaction may remain limited or delayed. When digital leaders consciously foster a supportive climate that honors employee autonomy during technological disruptions, they shift the exchange from a transactional mandate into a relational partnership, directly fueling organizational trust. It is this emergent trust rather than the cold mechanical deployment of digital tools that employees reciprocate through positive psychological feedback, establishing the full mediation mechanism observed in our model. Therefore, the impact of digital leadership on job satisfaction is expected to occur not directly, but rather through mediating variables. Research findings corroborate this, showing that organizational trust fully mediates this relationship. Within this complex interplay, organizational trust emerges as a vital psychological buffer that neutralizes the adverse effects of digital fatigue. The significant and positive impact of digital leadership on organizational trust clearly reveals the decisive role of leadership behaviors in shaping employee perceptions. It is stated that leaders’ capacity to build trust is a critical element in the digitalization process, and that trust is one of the fundamental building blocks of effective digital leadership (Abbu et al., 2022). When public sector personnel develop a strong baseline of trust in their institution and leaders, administrative demands and digital mandates are no longer perceived as tools for invasive surveillance or overwhelming workload. Instead, under a climate of trust, these technological changes are interpreted as supportive organizational investments designed to facilitate employee enablement and empowerment. Consequently, this psychological safety net dissolves the anxieties tied to technostress, allowing the visionary and transformative aspects of digital leadership to be successfully transmitted into enhanced job satisfaction. However, the strong and positive effects of leadership behaviors on organizational trust are also supported by numerous empirical and meta-analytic studies (Uslu and Oklay, 2015). In the context of digital leadership, communication, teamwork, and trust are cited as fundamental elements; digital leaders foster trust by sharing information transparently, establishing open communication channels, and involving employees in processes (López-Figueroa et al., 2025). Similarly, the literature emphasizes that a leader’s fair, consistent, and supportive behavior strengthens employees’ perception of trust (Pillai et al., 1999). In this context, digital leadership can be considered not only a tool for managing technological transformation but also a mechanism that determines the quality of organizational relationships. Importantly, while the structural path from digital leadership to organizational trust is potent and significant, the relatively low $R^2$ value obtained for the trust construct merits an explicit theoretical contextualization. In PLS-SEM modeling, a lower variance explanation for an endogenous mediator does not signals structural invalidity, but rather underscores the multifaceted and macro-level architecture of the construct in question. As comprehensively argued by Mayer et al. (1995), organizational trust is a complex, multi-referent phenomenon heavily dependent upon variables outside the micro-behaviors of an immediate leader such as institutional history, structural justice, job security, and socio-economic prestige (Mayer et al., 1995). Because these macro-organizational factors were naturally outside the scope of our specific leadership-centric framework, a low $R^2$ for trust is theoretically sound and empirically expected (Dirks and Ferrin, 2001). This outcome provides a compelling empirical revelation for public administration: while digital leadership is certainly not the sole omnibus determinant of complete institutional trust, its significant path coefficient proves that digital leadership serves as an essential, high-leverage strategic catalyst an ignition key that activates relational trust within digitally transforming public organizations. The strong influence of organizational trust on both job performance and job satisfaction is one of the most striking findings of the research. It is known that employees in organizations where a trusting environment prevails exhibit higher motivation, identify more strongly with organizational goals, and achieve higher performance (Huff and Kelley, 2003). Numerous studies in the literature examine the relationship between organizational trust and job satisfaction. Chathoth et al. (2007) found that organizational trust positively affects job satisfaction and that an increase in perceived trust significantly increases job satisfaction (Chathoth et al., 2007). Similarly, Akkoç et al. (2012) and Williams (2005) stated that organizational trust is strongly and positively related to different dimensions of job satisfaction (Williams, 2005; Niemi, 2025; Huff and Kelley, 2003; Plokha and Murzha, 2025; Akkoç et al., 2012). A study conducted in Türkiye also found significant relationships between organizational trust and job satisfaction (Top et al., 2012). The impact of organizational trust on job performance is also supported in the literature. A study conducted with 279 participants at a train manufacturing plant in northern China found that perceptions of organizational trust positively affected employee performance and that top management played a critical role in building trust (Ning et al., 2007). Similarly, a study conducted in Bangladesh with 395 employees found that job satisfaction and organizational commitment were positively correlated with both individual and collective employee performance (Lovely et al., 2019). When these findings are considered together, it can be said that, within the context of digital leadership, leadership behaviors can indirectly influence both job performance and job satisfaction by increasing organizational trust.

## 5 Conclusion

The study’s mediation results reveal that organizational trust partially mediates the effect of digital leadership on job performance and fully mediates its effect on job satisfaction. This situation shows that performance is influenced by both direct structural and technological factors, as well as socio-psychological factors; whereas job satisfaction is largely based on psychological and perceptual processes. In other words, while digital leadership can directly impact employee performance, it first needs to build trust to effectively influence job satisfaction.

### 5.1 Practical recommendations for public sector administrators

The empirical boundaries of the mediation model specifically, that organizational trust *partially* mediates job performance but *fully* mediates job satisfaction provide highly specific, actionable pathways for digital leaders within the Ministry of Youth and Sports:

1. Leveraging the Direct Path to Performance via Open Digital Dashboards: Since our findings demonstrate that digital leadership retains a significant direct impact on job performance (partial mediation), digital leaders can immediately drive task efficiency through structural and technological interventions. The ministry should deploy centralized, open digital dashboards to track nationwide youth projects and sports information systems. Because performance is driven directly by technological structure, real-time access to clear project timelines and resource metrics directly optimizes public personnel’s task execution by removing administrative ambiguities.
2. Addressing Full Mediation through Structured Feedback Loops: Crucially, because digital leadership has a non-significant direct effect on job satisfaction and relies entirely on organizational trust (full mediation), digital leaders cannot force employee satisfaction through technological mandates alone. To cultivate this indispensable trust within the ministry’s rigid bureaucratic culture, leaders must implement structured, non-punitive digital feedback loops. Automated, anonymous pulse surveys within the internal network should be established, allowing personnel to safely report digital friction or workload anxieties. Trust must be engineered first, as it is the sole psychological pipeline through which digital leadership translates into job satisfaction.
3. Establishing “Digital Borders” as a Trust-Building Asset: To prevent technostress from eroding the trust that fully fuels job satisfaction, the ministry must establish clear digital borders, such as formalized “Right to Disconnect” protocols. Digital leaders should set strict guidelines regarding off-hours communication. Protecting public sector employees from constant digital availability pressure demonstrates institutional care. This structural protection builds the foundational organizational trust required to unlock positive emotional evaluations and genuine job satisfaction among personnel during digital migration.

### 5.2 Limitations of the research

The study sample is limited to public employees working in the provinces of Kırşehir, Nevşehir, Yozgat, and Aksaray. This limits the generalizability of the findings to different sectors and cultural contexts. It is recommended that future studies include samples from the private sector and different countries.

The data is based on participants’ self-reports. Therefore, the responses given may not fully reflect actual behavior. It is recommended that future research utilize multiple data sources, such as management evaluations or performance records.

The study has a cross-sectional design. Therefore, the relationships between variables cannot be evaluated temporally. It is recommended that longitudinal research designs be used in future studies.

This study only examined the mediating role of organizational trust. However, variables such as organizational commitment, psychological empowerment, perceived workload, and burnout may also be influential in the model.

Including different mediating and moderating variables in the model in future research will contribute to a more comprehensive explanation of the relationships.

## Data Availability

The original contributions presented in the study are included in the article/Supplementary material, further inquiries can be directed to the corresponding author.
