# Supplementary material for: The mediating role of organizational trust in the impact of digital leadership on job performance and job satisfaction
Source: Front Psychol. 2026 Aug 13;17:1879796. doi: 10.3389/fpsyg.2026.1879796 (PMC13520208; doi:10.3389/fpsyg.2026.1879796)
Supplement: Supplementary file 1 [file Table_2.DOCX]

**The Medıatıng Role Of Organızatıonal Trust In The Impact Of Dıgıtal Leadershıp On Job Performance And Job Satısfactıon**

**Socio-Demographic Information**

**Gender	:** (	) Male	(	) Woman

**Marial Status	:** (	) Married	(	) Single

**Age	:** (	) 18-30	(	) 31-40	(	) 41-50	(	) 51-60	(	) 61 and over

**Graduation	:** (	) High School and Below	(	) Associate Degree	(	) University	(	) Postgraduate

**Eqperience Period :** ( )Up to 5 years	( )6-10 Year	( )11-15 Year	( )16-20 Year	( )21 Year and over

**Monthly Incom	:** (	) 20.001 - 40.000 TL	(	) 40.001 - 60.000 TL	(	) 60.001 - 80.000 TL

(	) 80.001 - 100.000 TL	(	) 100.001 TL and over

**DIGITAL LEADERSHIP SCALE**

|  | **I Strongly Disagree** | **I Disagree** | **I am undecided** | **I agree** | **I Strongly Agree** |
| --- | --- | --- | --- | --- | --- |
| **1. I find using digital tools fun.** |  |  |  |  |  |
| **2. I can say that I am an expert in digital devices and applications.** |  |  |  |  |  |
| **3. I constantly follow digital developments.** |  |  |  |  |  |
| **4. I am leading the digital transformation of the unit or organization I work for.** |  |  |  |  |  |
| **5. I guide my colleagues on digital transformation.** |  |  |  |  |  |
| **6. I have a clear understanding of the structures and processes needed for digital transformation.** |  |  |  |  |  |

**JOB SATISFACTION SCALE**

|  | **I Strongly Disagree** | **I Disagree** | **I am undecided** | **I agree** | **I Strongly Agree** |
| --- | --- | --- | --- | --- | --- |
| **1. I enjoy my job.** |  |  |  |  |  |
| **2. I find happiness in my work.** |  |  |  |  |  |
| **3. I am satisfied with my current job.** |  |  |  |  |  |
| **4. I enjoy my job.** |  |  |  |  |  |
| **5. Time passes well at work.** |  |  |  |  |  |

**JOB PERFORMANCE SCALE**

|  | **I Strongly Disagree** | **I Disagree** | **I am undecided** | **I agree** | **I Strongly Agree** |
| --- | --- | --- | --- | --- | --- |
| **1. Görevlerimi tam zamanında tamamlarım.** |  |  |  |  |  |
| **2. İş hedeflerime fazlasıyla ulaşırım.** |  |  |  |  |  |
| **3. Sunduğum hizmet kalitesinde standartlara fazlasıyla ulaştığımdan eminim** |  |  |  |  |  |
| **4. Bir problem gündeme geldiğinde en hızlı şekilde çözüm üretirim.** |  |  |  |  |  |

**ORGANIZATIONAL TRUST SCALE**

|  | **I Strongly Disagree** | **I Disagree** | **I am undecided** | **I agree** | **I Strongly Agree** |
| --- | --- | --- | --- | --- | --- |
| **1. I trust the job skills of my colleagues.** |  |  |  |  |  |
| **2. My colleagues are honest and trustworthy.** |  |  |  |  |  |
| **3. My colleagues are dedicated, and I don't hesitate to ask for their help when I need it.** |  |  |  |  |  |
| **4. I feel free to share all my work-related thoughts with my colleagues.** |  |  |  |  |  |
| **5. I trust that my colleagues will keep the promises they make to me.** |  |  |  |  |  |
| **6. My colleagues will not harm me for their own benefit.** |  |  |  |  |  |
| **7. In general, my colleagues are quite trustworthy.** |  |  |  |  |  |
| **8. My manager has the knowledge to monitor and improve my work.** |  |  |  |  |  |
| **9. My manager is always there to help when I need it.** |  |  |  |  |  |
| **10. I trust my manager not to tell anyone else about the information I've shared with them.** |  |  |  |  |  |
| **11. I can discuss any of my thoughts with my manager because I know they won't use them against me.** |  |  |  |  |  |
| **12. I have complete confidence that my manager will keep the promises he/she made to me.** |  |  |  |  |  |
| **13. I have full confidence that the right decisions will be made regarding the future of our institution.** |  |  |  |  |  |
| **14. Everything in this institution is conducted transparently and in accordance with the law; there are no secret practices.** |  |  |  |  |  |
| **15. The institution fairly addresses our personal rights.** |  |  |  |  |  |
| **16. The institution provides support to its employees in all matters.** |  |  |  |  |  |
| **17. I am not hesitant to develop suggestions and express my opinions regarding institutional policies.** |  |  |  |  |  |
